# Microwave-Assisted Synthesis of Cinnamyl Long Chain Aroma Esters

**DOI:** 10.3390/molecules200610594

**Published:** 2015-06-08

**Authors:** Marta Worzakowska

**Affiliations:** Department of Polymer Chemistry, Faculty of Chemistry, Maria Curie-Skłodowska University, Gliniana 33 Street, 20-614 Lublin, Poland; E-Mail: marta.worzakowska@poczta.umcs.lublin.pl; Tel./Fax: +48-81-524-2251

**Keywords:** 3-phenylprop-2-en-1-ol, esters, microwave irradiation

## Abstract

Cinnamyl long chain aroma esters were prepared by using the conventional and microwave-assisted methods. The esterification reaction of naturally occurring 3-phenyl-prop-2-en-1-ol and different chain lengths acidic and diol reagents was carried out at the temperature of 140 °C under solvent free conditions. As acidic reagents, oxolane-2,5-dione, oxane-2,6-dione, hexanedioic acid and decanedioic acid were applied. Ethane-1,2-diol and 2,2ʹ-[oxybis(2,1-ethandiyloxy)]diethanol were used as diol reagents. The synthesis of high molecular mass cinnamyl esters under conventional method conditions requires a long time to obtain high yields. The studies confirm that by using microwave irradiation, it is possible to reduce the reaction times to only 10–20 min. The structures of prepared esters were confirmed on the basis of FTIR, ^1^H-NMR and ^13^C-NMR. In addition, the newly obtained cinnamyl long chain esters were tested for their thermal properties. The TG studies proved the high thermal resistance of the obtained esters under inert and oxidative conditions.

## 1. Introduction

Aroma substances are one of the most important classes of organic compounds, used in many branches of industries like food, pharmaceutical, cosmetic, chemical, detergent, *etc.* [1,2,3,4]. They are volatile compounds with specific odors which are able to interact with human olfactory or taste receptors creating sensory impressions and perceptions [2]. Aroma compounds include many groups of natural, Nature-identical and artificial compounds such as hydrocarbons, alcohols, phenols, aldehydes, acetals, ketones, acids, esters, lactones, *etc.* [1,2,3,4,5,6,7,8,9]. Among them, flavors and fragrances are the two main groups with regard to their applications and sensory interactions. In general, flavors affect both the sense of smell and taste and due to this they are commonly utilized as aroma compounds for food and beverage products. However, fragrances affect only the sense of smell and are consequently mainly applied as aroma compounds in cosmetic, perfume and chemical industries [1,2,3,4,5,10].

Natural aroma compounds are mainly obtained from natural plant and fruit sources by physical methods like extraction, distillation, maceration or expression processes or by enzymatic and microbial methods. However, active substances often occur in minor quantities or as bound forms in plant materials. As a result, low yields of the desired aroma compounds and high isolation and purification cost occur [1,2,3,4,5,11]. Due to this, those preparation methods are economically unfavourable for global consumption of aroma compounds, and most of the valuable Nature-identical or artificial aromas are obtained through chemical synthesis methods [2,3,4,12,13,14,15].

Organic esters are important compounds in organic chemistry since they have a wide range of applications. They are applied as intermediate compounds to the synthesis of drugs and chemicals. Organic esters are utilized as plasticizers, solvents, lubricants, aroma compounds, *etc.* [16,17,18,19]. Many synthetic aroma esters can be conveniently prepared through the esterification process of a carboxylic acid and an alcohol in the presence of a strong mineral acid, the esterification process of an alcohol and an acid chloride in the presence of an amine as a catalyst or transesterification reactions [20,21] However, those reactions often require a large excess of substrates, high temperatures and toxic solvents in order to obtain a reasonable yield of the final product. In many cases, the use of strong mineral acids as catalysts of the esterification process can cause a rearrangement of the substrates and thus the creation of undesirable side products. This drastically reduces the efficiency of the synthesis and increases the production costs. Recently, we have proposed the use of butylstannoic acid as a catalyst in the synthesis of neryl, geranyl, citronellyl and cinnamyl diesters [22,23,24,25] and neryl and geranyl long chain esters [26,27]. This resulted in the preparation of aroma esters for cosmetic, laundry and perfume applications in high yield and purity under mild, solvent-free conditions. However, as was proved, those reactions required a long time and thus high energy consumption to obtain the end products in high yields. This significantly raises their production cost. Because of this, in the present paper we propose the application of the microwave heating technique for the synthesis of novel cinnamyl long chain aroma esters. The influence of microwave heating on the reaction time and reaction yield was evaluated and compared to the conventional heating conditions. In addition, the thermal properties of the prepared esters were evaluated by means of thermogravimetric analysis (TG).

## 2. Results and Discussion

### 2.1. Preparation of Cinnamyl Long Chain Esters

**Figure 1:**
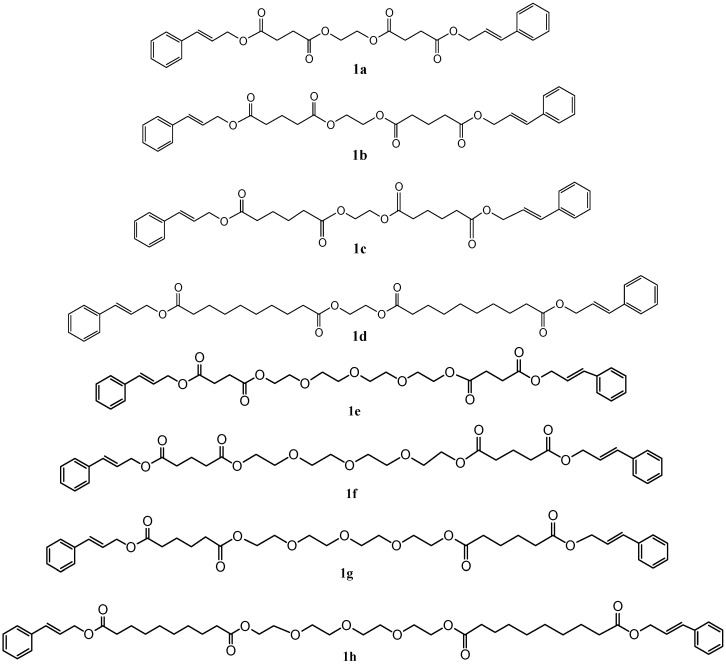
The structure of esters.

**Scheme 1:**
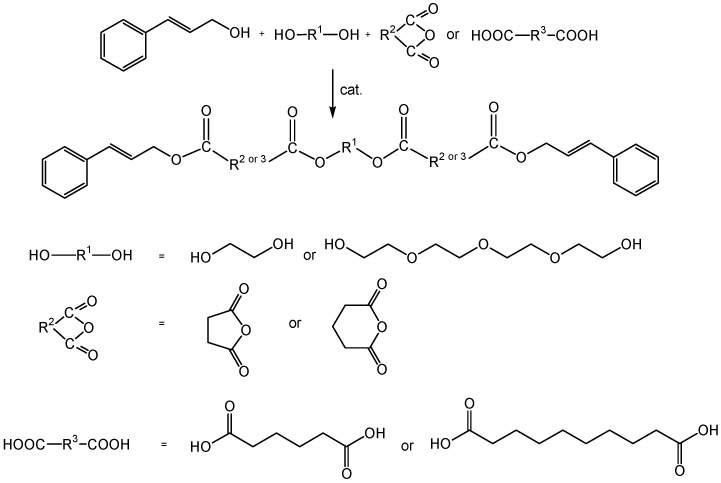
The reaction route.

The butylstannoic acid catalysed esterification reactions of stoichiometric ratios of 3-phenylprop-2-en-1-ol, suitable aliphatic acidic components (oxolane-2,5-dione, oxane-2,6-dione, hexanedioic acid or decanedioic acid) and diol component (ethane-1,2-diol or 2,2ʹ-[oxybis(2,1-ethandiyloxy)]diethanol) afforded novel, long chain aroma esters with an intensive, balsamic odor. The reactions were carried out at 140 °C under solvent-free conventional heating or microwave irradiation conditions. The reaction scheme and the structures of the prepared esters are presented in Scheme 1 and Figure 1, respectively.

The spectroscopic data were in good agreement with the proposed structures of the prepared esters (Experimental Section). Cinnamyl long chain esters are liquids, insoluble in water but soluble in organic solvents, with densities ranging from 1.0925 g/cm^3^ to 1.1250 g/cm^3^, viscosities ranging from 620 mPas to 965 mPas and refractive indexes higher than 1.5350 (Experimental Section). It is interesting to note that conventional heating and microwave irradiation heating resulted in comparable reaction yields (Table 1), the main difference being related to the time necessary to get a high yield. 

**Table 1:** The reaction time and reaction yields obtained by using the conventional and microwave assisted methods.

Compound	Reaction Time		Reaction Yield (%)	
	Conventional Method	Microwave Assisted Method	Conventional Method	Microwave Assisted Method
**1a**	30 h	10 min	90	90
**1b**	37 h	12 min	91	90
**1c**	42 h	14 min	91	92
**1d**	51 h	17 min	92	92
**1e**	39 h	12 min	90	91
**1f**	46 h	15 min	91	92
**1g**	58 h	18 min	92	90
**1h**	65 h	20 min	93	93

In order to obtain such a yield of the final products, the conventional esterification process needs a significantly longer time than the microwave assisted process. Reaction yields higher than 90% was obtained under conventional conditions after 30–65 h, whereas microwave irradiation allows reducing the synthesis time significantly, as only between 10 and 20 min are needed to prepare the final products with high purity. The results obtained are in accordance with our previous studies, where the influence of microwave irradiation on the synthesis of neryl long chain esters was evaluated [28]. As is clearly visible, the synthesis of esters **1a**–**1d** requires less time to obtain a high reaction yield than that of esters **1e**–**1h**. In addition, as the aliphatic chain length of acidic substrate is increased, the synthesis needs more irradiation time to be finished. It can be suspected that the differences are directly connected with the polarity of substrates used for the preparation of cinnamyl long chain esters [28,29,30,31].

### 2.2. Thermal Properties

The thermal properties of esters **1a**‒**1h** have been studied under inert and oxidative conditions using thermogravimetric analysis (TG, Table 2). The studies show that long chain esters derivatives of 3-phenylprop-2-en-1-ol are thermally stable up to 200 °C under both atmospheres. The beginning of their decomposition is observed when the temperature is higher than 225–252 °C for esters **1a**–**1d** and higher than 215–230 °C for esters **1e**–**1h**. It is worth noting that with the increase of the acidic aliphatic compound chain length, the thermal stability of esters becomes higher. In addition, the use of ethane-1,2-diol for the ester synthesis affords final products with better thermal stability as compared to esters prepared in the presence of 2,2ʹ-[oxybis(2,1-ethandiyloxy)]diethanol). Another observation is the slightly lower thermal resistance of esters under air than under inert atmosphere. Meanwhile, completely different decomposition pathways of esters under inert and oxidative conditions are observed. Under inert conditions, all the prepared esters decompose in one main stage with T_max_ values ranging from 360 °C to 417 °C (Table 2). This decomposition stage is directly connected with the breaking of some bonds, release of functional groups and ester chain rupture [22,23,24,25,26,27,32,33]. However, under oxidative conditions, the decomposition of esters takes places in two main stages with T_max_ values higher than 350 °C and 520 °C, respectively. The first decomposition stage is the result of the complex of simultaneous reactions including the breakage of some bonds (e.g., ester bonds, C-C bonds) and the free radical reactions of primary formed decomposition volatile products with oxygen, which leads to the creation of different volatile products and a residue. The second decomposition stage observed at temperatures higher than 520 °C may be associated with the oxidation process of the formed char residue [22,23,24,26,28].

**Table 2:** Thermal properties of esters under inert and oxidative conditions.

Compound	T_1%_/°C		T_max_/°C	
	Inert Conditions	Oxidative Conditions	Inert Conditions	Oxidative Conditions
**1a**	225	218	367	353/546
**1b**	230	220	371	361/532
**1c**	242	232	384	375/530
**1d**	252	244	402	384/533
**1e**	215	200	360	350/527
**1f**	220	208	367	352/538
**1g**	225	219	380	365/530
**1h**	230	225	417	379/524

T_1%_—the temperature where 1% of mass loss is observed, T_max_—DTG peak maximum temperature.

## 3. Experimental Section

### 3.1. General Information

ATR-FTIR was carried out with a TENSOR 27 spectrometer, equipped with a diamond crystal ATR (Bruker, Ettlingen, Germany). The spectra were recorded in the spectral range from 600 cm^−1^ to 4000 cm^−1^ with 64 scans per spectrum at a resolution of each spectrum of 4 cm^−1^. ^1^H- and ^13^C-NMR spectra were obtained on a Bruker-Avance 300 MSL spectrometer working at 300/75 MHz. Deuterated chloroform was used as the solvent. For ^1^H-NMR tetramethylsilane was used as an internal reference, while for ^13^C-NMR the solvent peak was used. The viscosity of esters was determined by a Brookfield DV-III rheometer (Stüttgart, Germany) at 25 °C. The density was measured with the use of a Gay/Lussac pycnometer at 25 °C. Refractive indexes were measured with a Carl Zeiss Jena refractometer (Jena, Germany) at 20 °C. The thermal properties of esters were determined on a STA 449 Jupiter F1 instrument (Netzsch, Selb, Germany). The samples were heated from 40 °C to 640 °C in Al_2_O_3_ crucibles with a heating rate of 10 °C·min^−1^. Helium and synthetic air (flow rate 40 mL/min) were used as furnance atmospheres.

### 3.2. Materials

3-Phenylprop-2-en-1-ol (cinnamyl alcohol, 98%) and oxane-2,6-dione (glutaric anhydride, 95%) were delivered by Fluka (Buchs, Switzerland). Ethane-1,2-diol (ethylene glycol, 99%), 2,2ʹ-[oxybis(2,1-ethandiyloxy)]diethanol (tetraethylene glycol, 99%), oxolane-2,5-dione (succinic anhydride, 99%), hexanedioic acid (adipic acid, 99%) and decanedioic acid (sebacic acid, 98%) were from Merck (Darmstadt, Germany). The butylstannoic acid used as a catalyst was obtained from Arkema Inc. (Philadelphia, PA, USA).

### 3.3. General Synthesis of Esters under Conventional and Microwave Conditions

Method A: In a typical procedure, cinnamyl alcohol (0.2 mol), acid anhydride or dicarboxylic acid (0.2 mol), diol (0.1 mol) and catalyst (0.15 wt %) were heated at 140 °C in a thermostatic oil bath under reduced pressure (200 mbar) for an appropriate reaction time (Table 1), in order to obtain the maximum reaction yield [26,27]. The reaction progress was monitored by thin layer chromatography (TLC).

Method B: The same reactants of method A were heated at 140 °C in a microwave oven (RM-800 multimode microwave reactor, Plazmatronika, Wrocław, Poland) under magnetic stirring for an appropriate reaction time (Table 1). The reaction progress was monitored by TLC. After the microwave reaction was finished, the raw product was cooled, dissolved in ethyl ether, dried over magnesium sulphate and then distilled under reduced pressure to remove the solvent [28].

*Dicinnamyl O*,*O′-ethane-1*,*2-diyl disuccinate* (**1a**). Formula C_28_H_30_O_8_, (calc. mass 494.540); refractive index (n_D20_) 1.5350; viscosity (ƞ_25 °C_) 620 mPas; density (d_25 °C_) 1.0955 g/cm^3^; ^1^H-NMR (δ ppm) 2.66–2.70 (d, 8H), 4.28 (bs, 4H), 4.72–4.78 (d, 4H), 6.20–6.32 (m, 2H), 6.60–6.70 (d, 2H), 7.40–7.42 (d, 4H), 7.32–7.36 (m, 4H), 7.25–7.30 (m, 2H); ^13^C-NMR (δ ppm) 28.56 (4CH_2_), 62.05 (2OCH_2_), 65.11 (2OCH_2_), 122.25 (2=CH), 125.80 (2ArC), 127.85 (4ArC), 128.50 (4ArC), 134.20 (2=CH), 135.89 (2ArC), 171.82 (4C=O); IR (thin film, cm^−1^) 3030 (ν =C-H), 3060 (ν =C-H), 2949 (ν C-H), 2880 (ν C-H), 1730 (ν C=O), 1658 (ν C=C), 1600 (ν C=C_Ar_), 1589 (ν C=C_Ar_), 1492 (δ C-H), 1448 (δ C-H), 1382 (δ C-H in ester), 1350 (δ C-H in ester), 1313 (ν C-O), 1147 (ν C-O), 1030 (ν C-O), 1070 (ν C-O), 964(γ =C-H), 744 (γ C_Ar_-H), 692 (γ C_Ar_-H).

*Dicinnamyl O*,*O′-ethane-1*,*2-diyl diglutarate* (**1b**). Formula C_30_H_34_O_8_, (calc. mass 522.594); refractive index (n_D20_) 1.5365; viscosity (ƞ_25 °C_) 743 mPas; density (d_25 °C_) 1.0925 g/cm^3^; ^1^H-NMR (δ ppm) 1.98–2.01 (m, 4H), 2.35–2.45 (m, 8H), 4.28 (bs, 4H), 4.70–4.78 (d, 4H), 6.20–6.32 (m, 2H), 6.60–6.70 (d, 2H), 7.40–7.42 (d, 4H), 7.32–7.36 (m, 4H), 7.25–7.30 (m, 2H); ^13^C-NMR (δ ppm) 19.85 (2CH_2_), 32.64 (4CH_2_), 62.15 (2OCH_2_), 65.10 (2OCH_2_), 122.85 (2=CH), 126.50 (2ArC), 127.90 (4ArC), 128.50 (4ArC), 134.25 (2=CH), 135.89 (2ArC), 172.18 (4C=O); IR (thin film, cm^−1^) 3030 (ν =C-H), 3058 (ν =C-H), 2925 (ν C-H), 2856 (ν C-H), 1730 (ν C=O), 1658 (ν C=C), 1600 (ν C=C_Ar_), 1579 (ν C=C_Ar_), 1494 (δ C-H), 1452 (δ C-H), 1382 (δ C-H in ester), 1352 (δ C-H in ester), 1300 (ν C-O), 1166 (ν C-O), 1126 (ν C-O), 1097 (ν C-O), 964 (γ =C-H), 746 (γ C_Ar_-H), 692 (γ C_Ar_-H).

*Dicinnamyl O*,*O′-ethane-1*,*2-diyl diadipate* (**1c**). Formula C_32_H_38_O_8_, (calc. mass 550.648); refractive index (n_D20_) 1.5360; viscosity (ƞ_25 °C_) 780 mPas; density (d_25 °C_) 1.0985 g/cm^3^; ^1^H-NMR (δ ppm) 1.60–1.73 (m, 8H), 2.31–2.43 (m, 8H), 4.28 (bs, 4H), 4.70–4.78 (d, 4H), 6.20–6.32 (m, 2H), 6.60–6.70 (d, 2H), 7.40–7.42 (d, 4H), 7.32–7.36 (m, 4H), 7.25–7.30 (m, 2H); ^13^C-NMR (δ ppm) 23.85 (4CH_2_), 33.57 (4CH_2_), 62.18 (2OCH_2_), 65.11 (2OCH_2_), 122.90 (2=CH), 126.55 (2ArC), 127.85 (4ArC), 128.55 (4ArC), 134.27 (2=CH), 135.90 (2ArC), 172.85 (4C=O); IR (thin film, cm^−1^) 3032 (ν =C-H), 3057 (ν =C-H), 2947 (ν C-H), 2877 (ν C-H), 1724 (ν C=O), 1655 (ν C=C), 1600 (ν C=C_Ar_), 1581 (ν C=C_Ar_), 1494 (δ C-H), 1448 (δ C-H), 1384 (δ C-H in ester), 1363 (δ C-H in ester), 1309 (ν C-O), 1157 (ν C-O), 1137 (ν C-O), 1078 (ν C-O), 964 (γ =C-H), 744 (γ C_Ar_-H), 692 (γ C_Ar_-H).

*Dicinnamyl O*,*O′-ethane-1*,*2-diyl disebacate* (**1d**). Formula C_40_H_54_O_8_, (calc. mass 662.864); refractive index (n_D20_) 1.5390; viscosity (ƞ_25 °C_) 825 mPas; density (d_25 °C_) 1.0995 g/cm^3^; ^1^H-NMR (δ ppm) 1.22–1.33 (m, 16H), 1.55–1.62 (q, 8H), 2.25–2.35 (m, 8H), 4.18–4.22 (t, 4H), 4.70–4.78 (d, 4H), 6.20–6.32 (m, 2H), 6.60–6.70 (d, 2H), 7.40–7.42 (d, 4H), 7.32–7.36 (m, 4H), 7.25–7.30 (m, 2H); ^13^C-NMR (δ ppm) 24.95 (8CH_2_), 28.89 (4CH_2_), 34.11(4CH_2_), 63.25 (2OCH_2_), 65.08 (2OCH_2_), 122.92 (2=CH), 126.58 (2ArC), 127.88 (4ArC), 128.65 (4ArC), 133.87 (2=CH), 135.95 (2ArC), 173.48 (4C=O); ); IR (thin film, cm^−1^) 3031 (ν =C-H), 3058 (ν =C-H), 2935 (ν C-H), 2856 (ν C-H), 1735 (ν C=O), 1654 (ν C=C), 1598 (ν C=C_Ar_), 1581 (ν C=C_Ar_), 1496 (δ C-H), 1463 (δ C-H), 1450 (δ C-H), 1392 (δ C-H in ester), 1357 (δ C-H in ester), 1301 (ν C-O), 1163 (ν C-O), 1124 (ν C-O), 1068 (ν C-O), 1056 (ν C-O), 964 (γ =C-H), 744 (γ C_Ar_-H), 692 (γ C_Ar_-H).

*Dicinnamyl O*,*O′-2*,*2ʹ-[oxybis(2*,*1-ethandiyloxy)]diethanyl disuccinate* (**1e**). Formula C_33_H_42_O_11_, (calc. mass 614.688); refractive index (n_D20_) 1.5365; viscosity (ƞ_25 °C_) 665 mPas; density (d_25 °C_) 1.1205 g/cm^3^; ^1^H-NMR (δ ppm) 2.66–2.70 (d, 8H), 3.65–3.70 (t, 8H), 4.30–4.38 (t, 8H), 4.75–4.78 (d, 4H), 6.20–6.32 (m, 2H), 6.60–6.70 (d, 2H), 7.40–7.42 (d, 4H), 7.32–7.36 (m, 4H), 7.25–7.30 (m, 2H); ^13^C-NMR (δ ppm) 29.50 (4CH_2_), 63.11 (2OCH_2_), 65.15 (2OCH_2_), 68.90 (3OCH_2_), 70.58 (3CH_2_), 122.87 (2=CH), 126.56 (2ArC), 127.95 (4ArC), 128.35 (4ArC), 133.95 (2=CH), 135.90 (2ArC), 172.11 (4C=O); IR (thin film, cm^−1^) 3030 (ν =C-H), 3060 (ν =C-H), 2945 (ν C-H), 2869 (ν C-H), 1730 (ν C=O), 1660 (ν C=C), 1598 (ν C=C_Ar_), 1579 (ν C=C_Ar_), 1494 (δ C-H), 1448 (δ C-H), 1386 (δ C-H in ester), 1346 (δ C-H in ester), 1311 (ν C-O), 1151 (ν C-O), 1112 (ν C-O), 1068 (ν C-O), 1031 (ν C-O), 964(γ =C-H), 744 (γ C_Ar_-H), 692 (γ C_Ar_-H).

*Dicinnamyl O*,*O′-2*,*2ʹ-[oxybis(2*,*1-ethandiyloxy)]diethanyl diglutarate* (**1f**). Formula C_35_H_46_O_11_, (calc. mass 642.742); refractive index (n_D20_) 1.5370; viscosity (ƞ_25 °C_) 770 mPas; density (d_25 °C_) 1.1250 g/cm^3^; ^1^H-NMR (δ ppm) 1.88–2.02 (m, 4H), 2.35–2.48 (m, 8H), 3.62–3.70 (t, 8H), 4.25–4.32 (t, 8H), 4.75–4.78 (d, 4H), 6.20–6.32 (m, 2H), 6.60–6.70 (d, 2H), 7.40–7.42 (d, 4H), 7.32–7.36 (m, 4H), 7.25–7.30 (m, 2H); ^13^C-NMR (δ ppm) 19.87 (2CH_2_), 32.80 (4CH_2_), 63.18 (2OCH_2_), 65.12 (2OCH_2_), 68.85 (3OCH_2_), 70.6 (3CH_2_),122.92 (2=CH), 126.60 (2ArC), 127.90 (4ArC), 128.40 (4ArC), 133.90 (2=CH), 135.90 (2ArC), 172.15 (4C=O); IR (thin film, cm^−1^) 3032 (ν =C-H), 3060 (ν =C-H), 2964 (ν C-H), 2924 (ν C-H), 2860 (ν C-H), 1730 (ν C=O), 1670 (ν C=C), 1598 (ν C=C_Ar_), 1579 (ν C=C_Ar_), 1494 (δ C-H), 1446 (δ C-H), 1377 (δ C-H in ester), 1340 (δ C-H in ester), 1300 (ν C-O), 1166 (ν C-O), 1126 (ν C-O), 1072 (ν C-O), 1026 (ν C-O), 964 (γ =C-H), 744 (γ C_Ar_-H), 692 (γ C_Ar_-H).

*Dicinnamyl O*,*O′-2*,*2ʹ-[oxybis(2*,*1-ethandiyloxy)]diethanyl diadipate* (**1g**). Formula C_37_H_50_O_11_, (calc. mass 670.796); refractive index (n_D20_) 1.5375; viscosity (ƞ_25 °C_) 800 mPas; density (d_25 °C_) 1.1820 g/cm^3^; ^1^H-NMR (δ ppm) 1.60–1.73 (m, 8H), 2.31–2.43 (m, 8H), 3.62–3.70 (t, 8H), 4.18–4.24 (t, 8H), 4.70–4.78 (d, 4H), 6.20–6.32 (m, 2H), 6.60–6.70 (d, 2H), 7.40–7.42 (d, 4H), 7.32–7.36 (m, 4H), 7.25–7.30 (m, 2H); ^13^C-NMR (δ ppm) 23.90 (4CH_2_), 33.60 (4CH_2_), 63.12 (2OCH_2_), 64.50 (2OCH_2_), 68.90 (3OCH_2_), 70.55 (3CH_2_), 122.90 (2=CH), 126.58 (2ArC), 127.89 (4ArC), 128.57 (4ArC), 134.10 (2=CH), 135.90 (2ArC), 172.88 (4C=O); IR (thin film, cm^−1^) 3026 (ν =C-H), 3059 (ν =C-H), 2947 (ν C-H), 2869 (ν C-H), 1726 (ν C=O), 1670 (ν C=C), 1598 (ν C=C_Ar_), 1579 (ν C=C_Ar_), 1494 (δ C-H), 1450 (δ C-H), 1386 (δ C-H in ester), 1361 (δ C-H in ester), 1300 (ν C-O), 1163 (ν C-O), 1132 (ν C-O), 1045 (ν C-O), 964 (γ =C-H), 744 (γ C_Ar_-H), 692 (γ C_Ar_-H).

*Dicinnamyl O*,*O′-2*,*2ʹ-[oxybis(2*,*1-ethandiyloxy)]diethanyl disebacate* (**1h**). Formula C_45_H_66_O_11_, (calc. mass 783.012); refractive index (n_D20_) 1.5390; viscosity (ƞ_25 °C_) 965 mPas; density (d_25 °C_) 1.1920 g/cm^3^; ^1^H-NMR (δ ppm) 1.22–1.33 (m, 16H), 1.55–1.62 (q, 8H), 2.28–2.35 (m, 8H), 3.62–3.70 (t, 8H), 4.18–4.25 (t, 8H), 4.70–4.78 (d, 4H), 6.20–6.32 (m, 2H), 6.60–6.70 (d, 2H), 7.40–7.42 (d, 4H), 7.32–7.36 (m, 4H), 7.25–7.30 (m, 2H); ^13^C-NMR (δ ppm) 24.85 (8CH_2_), 28.90 (4CH_2_), 34.15 (4CH_2_), 63.15 (2OCH_2_), 64.55 (2OCH_2_), 68.95 (3OCH_2_), 70.60 (3CH_2_), 122.90 (2=CH), 126.60 (2ArC), 127.90 (4ArC), 128.60 (4ArC), 133.90 (2=CH), 135.90 (2ArC), 173.50 (4C=O); IR (thin film, cm^−1^) 3033 (ν =C-H), 3064 (ν =C-H), 2954 (ν C-H), 2887 (ν C-H), 1728 (ν C=O), 1658 (ν C=C), 1596 (ν C=C_Ar_), 1577 (ν C=C_Ar_), 1496 (δ C-H), 1446 (δ C-H), 1380 (δ C-H in ester), 1359 (δ C-H in ester), 1311 (ν C-O), 1141 (ν C-O), 1068 (ν C-O), 964 (γ =C-H), 744 (γ C_Ar_-H), 692 (γ C_Ar_-H).

## 4. Conclusions

The studies reported herein confirmed that the use of microwave heating accelerates considerably the butylstannoic acid-catalysed esterification reactions of stoichiometric ratios of 3-phenylprop-2-en-1-ol with acidic and diol components having different aliphatic chain lengths in the structure as compared to conventional heating. Because of that, the microwave synthesis of novel, long chain aroma esters of 3-phenylprop-2-en-1-ol is attractive for practical applications since it allows obtaining esters in solvent free conditions, in high yield and shorter times. It significantly reduces the energy consumption and thus their preparation cost. In addition, the TG studies proved high thermal stability of the esters obtained, making them promising materials which can be applied as components or additives of products manufactured at high temperatures.
